# An Unusual Case of Pediatric Bilateral Congenital Optic Disc Pits With Unilateral High Myopia

**DOI:** 10.7759/cureus.12463

**Published:** 2021-01-03

**Authors:** Ravi Vaniyan, Aysha Falamarzi, Mohamed Yusuf Shaikh

**Affiliations:** 1 Ophthalmology, Royal Medical Services, Bahrain Defence Force Hospital, Riffa, BHR

**Keywords:** optic disc pit, high myopia, congenital, acquired, bilateral, pediatric, anisometropia

## Abstract

Optic disc pits are rare congenital abnormalities. They are unilateral in 85% of the affected individuals. Optic disc pits occurring in the highly myopic eyes of the older adults are supposed to be acquired due to mechanical expansion of the disc from the axial elongation. High myopia is also a well described association of the optic disc pits of the congenital nature.  We present a rare case of bilateral congenital optic disc pits in a 15-year-old girl having anisometropic unilateral axial myopia and the emmetropia in the fellow eye. This unique combination of the findings of unilateral high myopia in a child with bilateral congenital optic disc pits, to the best of our knowledge has not been described in the literature earlier. Our case demonstrates a scenario where two different causative factors for the optic disc pits may be present concurrently in the same instance.

## Introduction

First described in the late 19th century by Wiethe, optic disc pits (ODPs) are anomalous cavitations of the optic nerve head [1]. Generally described as congenital, optic disc pit is a rare disorder, sporadic in occurrence with the reported prevalence varying from 0.02% to 0.19%. [2-4]. Acquired optic disc pits also occur secondary to diseases such as glaucoma and myopia [2,3]. Congenital ODPs are generally unilateral but may be bilateral in up to 15% of patients [3]. Acquired ODPs can be bilateral in 21% to 48% of cases [5].  ODP is usually asymptomatic and may be detected incidentally during fundus examination. 

Certain ocular and systemic conditions are well-known associations for congenital ODPs. The examples include increased axial length, optic disc coloboma, megalopapilla, and certain rare systemic diseases such as Aicardi syndrome, Alagille syndrome, renal hypoplasia, and midline neurodevelopmental defects [2,6]. Myopia and glaucoma are known to be associated with acquired ODPs [3,7]. Optic disc pits associated with high myopia occurring in older adults are described variously in the literature either as congenital or acquired. We report an unusual case of bilateral optic disc pits in a pediatric setting with unilateral high myopia. Such a unique combination of the findings calls for a clear interpretation of the existing nomenclature for describing the optic disc pits.

## Case presentation

**Figure 1:**
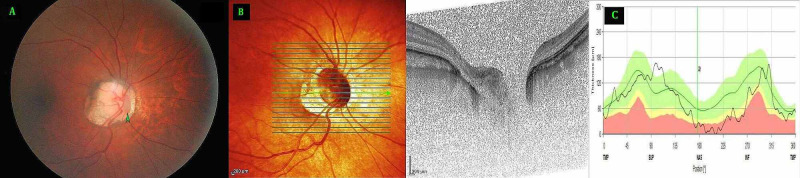
Right Eye (A) Fundus photo, (B) Optical coherence tomography (OCT) optic nerve head, (C) retinal nerve fibre layer distribution A: Fundus photo with arrow pointing at a vertically oval optic disc pit (ODP) in the nasal part of the optic disc with peripapillary myopic crescent and changes of generalized chorioretinal sclerosis. B: OCT optic nerve head showing the ODP located immediately next to the disc margin in the nasal portion of the optic disc in the zone four extending on to the adjacent zone three and five extending deeply beyond the level of lamina cribrosa. C: OCT optic nerve head showing the peripapillary retinal fiber layer distribution with a reduced thickness in the nasal and inferonasal segments.

**Figure 2:**
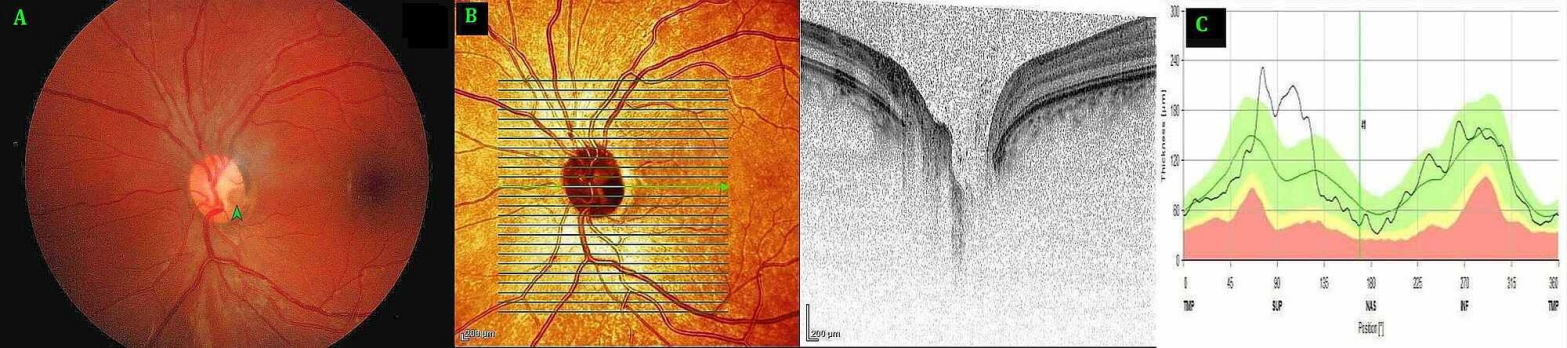
Left Eye (A) Fundus photo, (B) Optical coherence tomography (OCT) optic nerve head. (C) Distribution of retinal nerve fiber layer A: Fundus photo with arrow pointing at a round optic disc pit (ODP) in the inferotemporal part of the disc B: OCT optic nerve head showing the ODP located in the temporal part of the disc in zone one extending deeply beyond the level of lamina cribrosa. C: OCT optic nerve head showing a normal distribution of peripapillary retinal nerve fiber layer.

A 15-year-old girl reported to our clinic with history of long-standing poor vision in her right eye. She had no other eye symptoms and no recall of previous eye checkups. Her past history including perinatal history and her general health were unremarkable. She is the third child of a non-consanguineous marriage and her other three siblings and the parents are not known to have any systemic or ocular disease. Her uncorrected visual acuity in the right eye was 6/90, not improving beyond 6/30 with corrective glasses or pinhole. Her left eye had uncorrected visual acuity of 6/6. Cycloplegic refraction revealed a refractive error of -8.75 D sphere/-2 D cylinder at 151 degrees in the right eye and no error of refraction in the left eye. Anterior segment examination was within normal limits. Intraocular pressure was 16 mm of Hg in right eye and 17 mm of Hg in left eye by Goldmann’s applanation tonometry. Dilated fundus examination revealed in the right eye, a clearly noticeable vertically oval pit in the nasal part of the optic disc with peripapillary myopic crescent and changes of generalized chorioretinal sclerosis (Figure 1A). The emmetropic left eye revealed a prominent round looking pit at the infero-temporal part of the disc (Figure 2A). Macular area was clinically free from any abnormality in both the eyes.

Spectral Domain-Optical Coherence Tomography (Spectralis OCT+HRA, Heidelberg Engineering, Germany) was used to study the presence and extent of the pits (Figure 1B, 2B). The peripapillary retinal nerve fiber layer analysis showed nasal thinning in the right eye (Figure 1C, 2C). OCT study of macular region was unremarkable in both eyes. Visual field study with Humphrey field analyzer using 24-2 test did not reveal any specific visual field defects in either eye. Neuroimaging of brain and orbits revealed no abnormalities. Systemic and neurological evaluation was unremarkable as well. 

The mildly pale-looking myopic optic disc of the right eye showed a grey looking ODP that was vertically oval, with a surface measurement of about 1/2 of the disc diameter vertically and 1/5th horizontally. It was located immediately next to the disc margin in the nasal portion of the optic disc in zone four extending on to the adjacent zones three and five (Zones as described by Ohno-Matsui et al. [8]). In the emmetropic fellow left eye, the grey looking circularly shaped pit measuring just over 1/4th disc diameter, was located in the temporal part of the optic disc in the zone one. In both eyes the pits extended deeply beyond the level of lamina cribrosa. Macular region was unremarkable in both eyes. Keratometry readings using IOL Master (Zeiss IOL Master 500, Zeiss Medical Technology, Jena, Germany) were similar in both the eyes (right eye K1 43.77 / K2 45.86 D and left eye K1 44.29 D/ K2 45.49 D). Corneal pachymetry readings were 532 µ and 551 µ respectively in right and left eye. The axial length measurements however showed marked disparity (right eye 25.70 mm, left eye 22.17 mm). The optic disc size in the myopic right eye was larger, with disc area of 3.88 mm^2^ compared to 2.25 mm^2^ in the left eye. 

A diagnosis of bilateral congenital optic disc pits with unilateral high axial myopia with amblyopia was made and her condition remained stable over the further follow up of one year.

## Discussion

The prevalence of ODP is approximately 1:11,000. The majority of cases are thought to be congenital. However, acquired ODPs do occur secondary to glaucoma or myopia. Congenital ODPs are most often unilateral, but they can be bilateral in approximately 15% of cases. Of the acquired cases, in 21% to 48%, the ODPs are bilateral. Our case who is a 15-year young child is unusual, as it has bilateral optic disc pits but has high axial myopia present only in one eye. 

Optic disc pits associated with high myopia have been variously described in the literature as congenital or acquired [3,8]. Certain clinical features are known to be more commonly associated with the acquired compared to the congenital pits. These features by themselves including the OCT studies; however, cannot establish the true nature of the pits with certainty [8]. 

Congenital optic pits are known to differ from the acquired pits found in high myopia; in numbers, their location on the disc, laterality and in the prevalent sex distribution. Congenital ODPs are generally unilateral, mostly temporal in location, usually single with equal prevalence in both sexes [8]. Seventy percent of congenital ODPs are located in the inferotemporal segment of the optic disc, 20% centrally, and 10% in the other regions. Ophthalmoscopically, the congenital pits are generally round in shape. In contrast, the acquired pits of high myopia are slit-like and can be often difficult to discern without the aid of OCT and are located at superior or inferior edges. Occasionally they are observed in the peripapillary myopic conus area. Acquired pits of the disc may vary from one to seven in numbers, 21% to 48% of them occur bilaterally and twice as frequently in women [2,3,9]. In our case, in the myopic eye, there was only a single pit that was noticeably large and oval rather than slit-like and was located in the nasal part of the disc rather than at the vertical edges. The increased axial length and increased optic disc size are well-established associations of myopic ODPs [3]. Both these features were well evident in our case. 

There is no consensus on the embryologic origins of congenital ODPs either on the time of their formation or the tissues affected. The most widely accepted hypothesis proposes abnormal differentiation in neuroectodermal folds of the primitive papillae [2]. The acquired ODPs on the other hand are presumed to be due to the defects developing in lamina cribrosa as a result of stretching occurring in high myopia or advanced glaucoma. In highly myopic eyes, it has been hypothesized that the optic disc undergoes mechanical expansion to become megalodisc-like, which is accompanied by stretching and expanding of the lamina cribrosa leading ultimately to dehiscence of the lamina from the peripapillary sclera, with disruption of the overlying nerve fibers or herniation of these fibers into the defect [3]. Earlier researchers had shown the presence of membrane spanning the congenital but not the acquired pits. However, this was not confirmed in later studies [2,3]. No such membrane was seen on the OCT in our case either. 

The detection of optic disc pits is often coincidental and at times easily missed in the absence of appropriate OCT examinations [3]. High myopia is described in the literature as an association interestingly for both congenital and acquired optic disc pits. In a large series of the acquired myopic pits, the mean age of the acquired myopic pits was 52.8 years [3]. In another series of the congenital optic disc pits, the occurrence of congenital ODP in association with high myopia was documented at the age of 37 years [8]. Our patient is much younger presenting with high myopia and optic disc pit at an early age of 15 years.  

In terms of the laterality, the congenital optic disc pits are generally unilateral in occurrence. Our case is again unusual in a way that the bilateral optic disc pits are present in the presence of unilateral high myopia with the other eye being completely emmetropic. Such a combination, to the best of our knowledge, has not been described in the literature earlier. Interestingly in a 55-year-old man with bilateral retinitis pigmentosa inversa, having unilateral very high myopia, the occurrence of the pit was described unilaterally in the emmetropic eye rather than in the myopic eye [9].  

## Conclusions

High myopia is a well-described association of the optic disc pits of both acquired and congenital nature. Our case demonstrates a unique scenario where the two known causative factors for the optic disc pit formation may be concurrently present in the same instance.  

In conclusion, our case represents an unusual occurrence of bilateral congenital optic disc pits in a child with the unilateral association of high axial myopia, and to the best of our knowledge, such a combination of the findings has not been reported in the literature earlier. 
